# Supplementary material for: Computational and cellular studies reveal structural destabilization and degradation of MLH1 variants in Lynch syndrome
Source: eLife. 2019 Nov 7;8:e49138. doi: 10.7554/eLife.49138 (PMC6837844; doi:10.7554/eLife.49138)
Supplement: Transparent reporting form [file elife-49138-transrepform.docx]

***eLife’s* transparent reporting form**

We encourage authors to provide detailed information *within their submission* to facilitate the interpretation and replication of experiments. Authors can upload supporting documentation to indicate the use of appropriate reporting guidelines for health-related research (see [EQUATOR Network](http://www.equator-network.org/%20)), life science research (see the [BioSharing Information Resource](https://biosharing.org/)), or the [ARRIVE guidelines](http://www.plosbiology.org/article/info:doi/10.1371/journal.pbio.1000412) for reporting work involving animal research. Where applicable, authors should refer to any relevant reporting standards documents in this form.

If you have any questions, please consult our Journal Policies and/or contact us: [editorial@elifesciences.org](mailto:editorial@elifesciences.org).

**Sample-size estimation**

- You should state whether an appropriate sample size was computed when the study was being designed
- You should state the statistical method of sample size computation and any required assumptions
- If no explicit power analysis was used, you should describe how you decided what sample (replicate) size (number) to use

Please outline where this information can be found within the submission (e.g., sections or figure legends), or explain why this information doesn’t apply to your submission:

**Experiments:**

No power analyses were performed. As we have done previously, experiments were performed between three and five times.

**Computation:**

FoldX calculations of changes in protein stability were carried out in 5 independent trials, averages are reported. This number is the highest of what is recommended in the FoldX manual for the BuildModel command (<http://foldxsuite.crg.eu/command/BuildModel>).

Replicate consideration does not apply for calculation of conservation and co-variation.

**Replicates**

- You should report how often each experiment was performed
- You should include a definition of biological versus technical replication
- The data obtained should be provided and sufficient information should be provided to indicate the number of independent biological and/or technical replicates
- If you encountered any outliers, you should describe how these were handled
- Criteria for exclusion/inclusion of data should be clearly stated
- High-throughput sequence data should be uploaded before submission, with a private link for reviewers provided (these are available from both GEO and ArrayExpress)

Please outline where this information can be found within the submission (e.g., sections or figure legends), or explain why this information doesn’t apply to your submission:

**Experiments:**

We define biological repeats as experiments where transfections were performed separately.

Fluorescence microscopy was carried out in five biological repeats with each based on 200 and 1000 cells (legend to Fig. 2B). Quantifications of Western blots were in all cases based on three biological repeats (legends to Fig. 3BD, 4BEF, 5BD).

**Computation:**

We calculated averages of FoldX ∆∆Gs; these can be found as a supplemental file.

**Statistical reporting**

- Statistical analysis methods should be described and justified
- Raw data should be presented in figures whenever informative to do so (typically when N per group is less than 10)
- For each experiment, you should identify the statistical tests used, exact values of N, definitions of center, methods of multiple test correction, and dispersion and precision measures (e.g., mean, median, SD, SEM, confidence intervals; and, for the major substantive results, a measure of effect size (e.g., Pearson's r, Cohen's d)
- Report exact p-values wherever possible alongside the summary statistics and 95% confidence intervals. These should be reported for all key questions and not only when the p-value is less than 0.05.

Please outline where this information can be found within the submission (e.g., sections or figure legends), or explain why this information doesn’t apply to your submission:

**Experiments:**

For the fluorescence microscopy quantifications the errors indicate the SEM (n=5). No statistical tests were performed. For Western blotting, the error bars indicate the standard deviation. No statistical tests were performed.

**Computation:**

For the comparison of ∆∆Gs and evolutionary sequence energies, we calculate average and SEM within each group. The underlying data can be found in the supplemental files. We do not analyze SD within the FoldX calculation for each variant as there is not validated use for or interpretation of these numbers. Instead, the protocol that we use involves repeated computation and reporting just the average of these.

(For large datasets, or papers with a very large number of statistical tests, you may upload a single table file with tests, Ns, etc., with reference to sections in the manuscript.)

**Group allocation**

- Indicate how samples were allocated into experimental groups (in the case of clinical studies, please specify allocation to treatment method); if randomization was used, please also state if restricted randomization was applied
- Indicate if masking was used during group allocation, data collection and/or data analysis

Please outline where this information can be found within the submission (e.g., sections or figure legends), or explain why this information doesn’t apply to your submission:

**Experiments & Computations:**

Assignment of pathogenicity was taken from the ClinVar database as described in the manuscript. Assignment of Dominant Mutator Effect (DME) was calculated from the Takahashi 2007 paper, where each variant was tested in 3 different functional assays (see Methods section for further details).

**Additional data files (“source data”)**

- We encourage you to upload relevant additional data files, such as numerical data that are represented as a graph in a figure, or as a summary table
- Where provided, these should be in the most useful format, and they can be uploaded as “Source data” files linked to a main figure or table
- Include model definition files including the full list of parameters used
- Include code used for data analysis (e.g., R, MatLab)
- Avoid stating that data files are “available upon request”

Please indicate the figures or tables for which source data files have been provided:

**Experiments & Computations:**

All FoldX ∆∆Gs are provided in the supplemental files. DME, ClinVar assignments, ∆∆Gs and evolutionary sequence energies for the variants discussed here are provided in the supplemental files.
